# Thyme Extract Alleviates High-Fat Diet-Induced Obesity and Gut Dysfunction

**DOI:** 10.3390/nu15235007

**Published:** 2023-12-04

**Authors:** Yu Ra Lee, Hye-Bin Lee, Mi-Jin Oh, Yoonsook Kim, Ho-Young Park

**Affiliations:** 1 Food Functionality Research Division, Korea Food Research Institute, Wanju-gun 55365, Republic of Korea; lyr@kfri.re.kr (Y.R.L.); l.hyebin@kfri.re.kr (H.-B.L.); mjoh@kfri.re.kr (M.-J.O.); kimyus@kfri.re.kr (Y.K.); 2 Department of Food Biotechnology, Korea National University of Science and Technology, Daejeon 34113, Republic of Korea

**Keywords:** *Thymus vulgaris* L., high-fat diet, obesity, obesity, gut dysfunction, metabolic endotoxemia

## Abstract

Prolonged intake of a high-fat diet (HFD) disturbs the composition of gut microbiota, contributing to the development of metabolic diseases, notably obesity and increased intestinal permeability. Thyme (*Thymus vulgaris* L.), an aromatic plant, is known for its several therapeutic properties. In this study, we explored the potential of thyme extract (TLE) to mitigate HFD-induced metabolic derangements and improve the gut environment. Eight-week-old C57BL/6 mice were administered 50 or 100 mg/kg TLE for eight weeks. Administration of 100 mg/kg TLE resulted in decreased weight gain and body fat percentage, alongside the regulation of serum biomarkers linked to obesity induced by a HFD. Moreover, TLE enhanced intestinal barrier function by increasing the expression of tight junction proteins and ameliorated colon shortening. TLE also altered the levels of various metabolites. Especially, when compared with a HFD, it was confirmed that 2-hydroxypalmitic acid and 3-indoleacrylic acid returned to normal levels after TLE treatment. Additionally, we investigated the correlation between fecal metabolites and metabolic parameters; deoxycholic acid displayed a positive correlation with most parameters, except for colon length. In contrast, hypoxanthine was negatively correlated with most parameters. These results suggest a promising role for thyme in ameliorating obesity and related gut conditions associated with a HFD.

## 1. Introduction

*Thymus vulgaris* L., a notable aromatic plant distributed worldwide, has extensive medicinal and culinary applications [1]. Fresh thyme has exceptional antioxidant content, which is superior to that of other herbs. The therapeutic properties of thyme are primarily found in its essential oil, which exhibits expectorant, antispasmodic, bactericidal, and anthelmintic characteristics [2]. Furthermore, Thymes have a history of traditional use in folk remedies, predominantly in the preparation of leaf infusions, which are used as expectorants, cough suppressants, cold remedies, and antimicrobial agents [3], and are also used in therapies for mental disorders, including anxiety and depression [4]. 

Mechanistically, thyme extract (TLE) has been suggested to inhibit TNF-α, IL-6, and other inflammatory cytokines. It can potentially play a protective role against detrimental outcomes of diseases [5] and can be utilized as a therapeutic agent [6]. In addition, an investigation into the antioxidant and anticancer activities of thyme in human breast cancer cells confirmed an increase in apoptosis [7]. Specifically, thyme extract notably reduced the synthesis and genetic expression of inflammatory mediators while enhancing these parameters for the anti-inflammatory cytokine, IL-10 [8]. In addition, thyme extract functions as an anti-inflammatory agent by scavenging nitric oxide radicals that affect the onset of inflammatory conditions and significantly inhibits the expression of iNOS mRNA [9]. A hydroalcoholic extract of thyme has been shown to exert regulatory effects on both acute and chronic pain in mice [10].

Thyme extract has also been reported to exhibit hepatoprotective effects [11]. Recently, the effects of TLE on lead toxicity were demonstrated; lead toxicity increased the levels of TNF-α, IL-1β, and IL-6, while decreasing those of IL-10 and IFN-γ. These findings suggest that TLE alleviated lead-induced stress in hepatic and renal tissues and exhibited potential as an immunomodulatory, antioxidant, and protective agent against lead toxicity [12].

Plants of the Thymes are significant medicinal herbs known for their antimicrobial properties and abundant active compounds [13]. Thyme essential oils exhibit antibacterial effects against Gram-positive bacteria, including *Streptococcus pyogenes* [14]. Furthermore, an 80% ethanol extract of thyme leaf exhibited antiviral activity against Newcastle disease virus [15]. The antioxidant activity of thyme phenolics is exceptionally high, possibly surpassing that of the widely recognized antioxidants such as butylated hydroxy toluene and α-tocopherol [16]. Thyme essential oil possesses robust radical-scavenging capability, making it a natural antioxidant [17].

Thyme extract also exhibits antispasmodic effects in addition to its anti-inflammatory and antimicrobial roles. A study revealed that thyme extract affected the contractile responses in the guinea pig ileum, potentially affecting the cholinergic and serotoninergic pathways [18]. Furthermore, the antispasmodic effects of the extract on the smooth muscles of the respiratory tract and intestines were also reported [19].

Thyme effectively upregulates the expression of tight junction proteins and exerts prebiotic effects [20]. Thyme extract was shown to enhance the composition of gut microbiota [21]. In a previous study, we established that molokhia leaves modulate the gut microbiota composition and enhance the intestinal barrier function [22]. Similarly, we demonstrated the production of metabolites beneficial for gut health by gellan gum [23]. The anti-obesity and gut health-promoting effects of thyme extract have not been sufficiently explored. To overcome this gap, in this study, we investigated the anti-obesity and gut-health promoting effects of TLE in high-fat diet (HFD)-fed obese mice. Additionally, we conducted a correlation analysis between metabolic disorder parameters and fecal metabolites to examine the changes in the metabolic profile of the extract. Our findings have important implications for the development of novel plant extracts to treat obesity and improve intestinal health.

## 2. Materials and Methods

### 2.1. Preparation of Water-Soluble TLE

*T. vulgaris* leaves, sourced from Jeollabuk-do, Republic of Korea, were dried and ground into a powdered form. The leaf powder was extracted with 20 volumes of distilled water at 80 °C for 3 h and the extract was filtered through a filter paper (No. 4, Whatman, Maidstone, UK). The extracts were concentrated using a vacuum rotary evaporator and freeze-dried to obtain TLE.

### 2.2. Animals

Male 7-week-old C57BL/6 mice (weighing 19.0–21.0 g) were obtained from Orient Bio, Inc. (Seongnam, Republic of Korea). All the mice were housed in a controlled room maintained at 22 ± 2 °C and 50 ± 10% relative humidity with a 12 h-12 h light-dark cycle. The animals were fed AIN-93G diet (Dyets, Bethlehem, PA, USA) and provided sterilized water. All the animal experiments were approved by the Korea Food Research Institutional Animal Care and Use Committee (approval number: KFRI-M-22038).

After a week of adaptation, 45 mice were randomly divided into the ND, HFD, PC, TLE50, and TLE100 groups (*n* = 9 per group). During the 8-week experimental period, the ND group received the AIN-93G diet, whereas the other groups received a 60% HFD (TD.06414, Harlan, Madison, WI, USA). The PC group was orally administered fructooligosaccharides (50 mg/kg/day), and the TLE50 and TLE100 groups were orally fed TLE (50 or 100 mg/kg/day). The ND and HFD groups were orally administered sterilized water. Body weight was recorded once a week and body composition, oral glucose tolerance, and intestinal permeability were analyzed at week 8. Three mice were housed in a single cage. Fecal samples were obtained by pooling feces from three mice, collected from a total of three cages, and analyzed. Fresh fecal samples were collected once at the 8th week and immediately stored in a deep freezer.

### 2.3. InAlyzer Analysis

The body composition of mice was assessed using dual-energy X-ray absorptiometry (InAlyzer; Medikors Inc., Seongnam, Republic of Korea). After administration of anesthesia, the mice were positioned within the InAlyzer scanning area for a complete body scan. The InAlyzer software (v.1.0.0.0) was used to capture images and to determine the body fat mass.

### 2.4. Biochemical Analysis

After a 12 h fasting period, the mice were humanely euthanized by isoflurane exposure, and blood samples were collected. Serum was isolated by centrifuging the blood at 1500× *g* for 15 min and was stored at −80 °C. Biochemical analyses of the serum and liver samples were conducted using commercial ELISA kits from various manufacturers: Thermo Fisher Scientific (endotoxin, A39552; insulin, EMINS, Waltham, MA, USA); Cusabio (alanine aminotransferase (ALT), CSB-E16539m; aspartate aminotransferase (AST), CSB-E12649m, Houston, TX, USA); Abcam (triglycerides (TG), ab65336; cholesterol, ab65390, Cambridge, UK); and R&D Systems (leptin, DY498-05; Minneapolis, MN, USA). Data were analyzed according to the manufacturer’s instructions. Insulin resistance was determined using the following formula:

Homeostatic model assessment for insulin resistance (HOMA-IR) = fasting insulin (µU/mL) × fasting glucose (mg/dL)/405

### 2.5. Oral Glucose Tolerance Test

At 8 weeks of treatment, mice that had been fasted for 12 h were administered an oral dose of 2 g glucose/kg body weight. Blood glucose levels were assessed at 0, 30, 60, 90, and 120 min after glucose administration using a glucometer (Accu-Chek; Roche Diagnostics, Indianapolis, IN, USA).

### 2.6. Colon Length and pH Measurement

Following euthanasia, the entire colon, spanning the region from the cecum to the rectum, was dissected, and its length was measured with a ruler as an indicator of inflammation [24]. The pH of the colon was assessed by flushing its contents with sterile water and measuring its pH with a pH meter (Orion Star A211; Thermo Fisher Scientific).

### 2.7. Assessment of Intestinal Permeability

At 8 weeks of treatment, mice were fasted for 6 h and then administered an oral dose of FITC-dextran (500 mg/kg). Blood samples were collected from the tail vein 2 and 5 h after the administration of FITC-dextran. Plasma was obtained by centrifuging the blood (1500× *g*, 15 min). Standards were established by serially diluting FITC-dextran in untreated plasma. Plasma fluorescence levels were measured using a microplate reader (Molecular Devices, Sunnyvale, CA, USA) at excitation and emission wavelengths of 485 and 535 nm, respectively.

### 2.8. Western Blotting

Total protein was extracted from the colon and quantified using an RC DC™ Protein Assay Kit (Bio-Rad, Hercules, CA, USA). Proteins were subsequently loaded onto 4–20% polyacrylamide gels and transferred onto polyvinylidene difluoride membranes. The membranes were incubated overnight at 4 °C with primary antibodies specific to zonula occludens-1 (ZO-1), occludin, claudin-1, and β-actin, purchased from Abcam (Cambridge, MA, USA). Thereafter, the membranes were incubated with secondary antibodies, and protein bands were detected by EZ-Western Lumi Femto Kit (DoGenBio Co., Ltd., Seoul, Republic of Korea) and visualized using a ChemiDoc XRS+ imaging system (Bio-Rad).

### 2.9. Metabolite Profiling

Feces samples (50 mg) were extracted with 750 µL of 50% MeOH, followed by three cycles of sonication for 10 min each on an ice bath. Subsequently, the homogenate was centrifuged (13,000× *g* at 4 °C for 10 min) and the supernatant was filtered through a PTFE syringe filter and subjected to liquid chromatography-tandem mass spectrometry (LC-MS/MS) analysis.

All MS/MS experiments were performed using a Xevo TQ MS instrument (Waters, Manchester, UK) with an ACQUITY UPLC BEH C18 column (1.7 μm, 2.1 × 100 mm). The flow rate was set at 0.3 mL/min, with 0.1% formic acid (FA) in water as solvent A and 0.1% FA in acetonitrile (ACN) as solvent B. The solvent gradient was as follows: 0–17.5 min, a linear increase from 5% to 95% B; 17.5–19 min, 95% to 89% B; 19–19.5 min, a linear decrease from 89% to 5% B; and 19.5–22 min, 5% B. The column oven was maintained at 40 °C, and the injection volume was 5 μL. The capillary, cone, and extractor voltages were set to 3 kV, 16 V, and 3 V, respectively. The source temperature, desolvation temperature, and desolvation gas flow rate were maintained at 150 °C, 400 °C, and 800 L/h, respectively. In Table 1, multiple reaction monitoring conditions used for the analysis of thirty-one metabolites are shown.

### 2.10. Statistical Analysis

Statistical analyses were performed using SPSS 20 (IBM Corp., Armonk, NY, USA). The statistical differences among various groups were calculated by one-way ANOVA followed by Tukey’s post hoc test. Simple correlation was analyzed using Spearman’s correlation by R studio (version 2023.09.1+494) and visualized using corrplot packages (version 0.92).

## 3. Results

### 3.1. Changes in Body and Liver Weight

**Figure 1:**
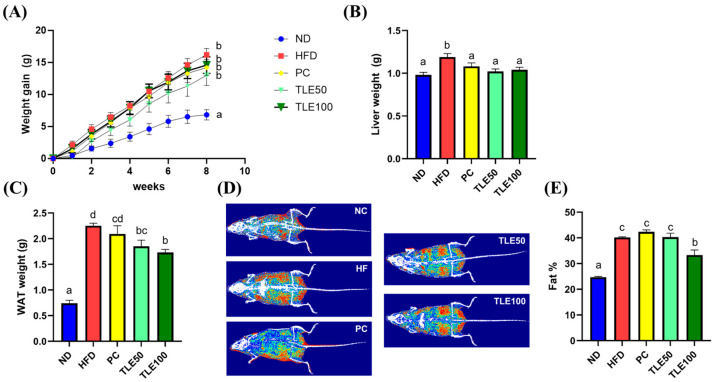
Effects of administration of thyme leaf extract (TLE) on the body, liver, and epididymal white adipose tissue (WAT) weights and body fat content in high-fat diet (HFD)-induced obese mice. Changes in (**A**) body weight gain, (**B**) liver weight, and (**C**) WAT weight; (**D**) representative images of body fat composition; (**E**) quantitation of body fat percent. The data are expressed as mean ± standard error of the mean (*n* = 9). Significant differences (*p* < 0.05) are indicated with different letters (a–d), and were determined using one-way ANOVA followed by Tukey’s post hoc test.

Mice in the HFD group exhibited a notable increase in body weight when contrasted with those in the ND group (Figure 1A). The liver weight showed a significant increase only in the HFD group, with no significant differences observed between the PC, TLE50, and TLE100 groups compared with the liver weight in the normal group (Figure 1B). At 8 weeks of treatment, the TLE 100 mg/kg group showed a notable reduction in the weight of the white adipose tissue compared with the HFD group (Figure 1C). The examination of body composition through the InAlyzer demonstrated a noteworthy elevation in fat percentage among overweight mice as opposed to that in ND mice (Figure 1D,E). Furthermore, the group administered 100 mg/kg TLE exhibited a significant reduction in fat percentage compared with the HFD group.

### 3.2. Effect of TLE on Serum Biochemical Parameters

**Figure 2:**
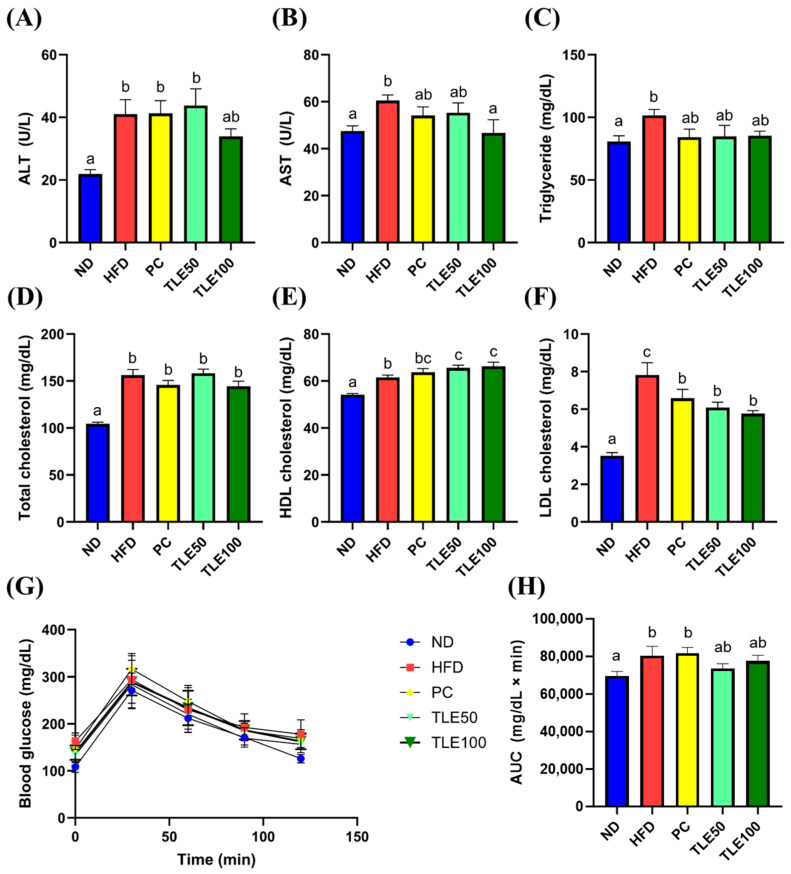
Effects of thyme leaf extract (TLE) on serum indicators of liver function, lipid parameters, and blood glucose tolerance level in high-fat diet (HFD)-induced obese mice. (**A**) Serum alanine aminotransferase (ALT); (**B**) serum aspartate aminotransferase (AST); (**C**) serum triglyceride; (**D**) serum total cholesterol; (**E**) serum HDL cholesterol; (**F**) LDL cholesterol; (**G**) blood glucose levels in the oral glucose tolerance test; (**H**) area under the curve (AUC) of blood glucose levels. The data are expressed as mean ± standard error of the mean (*n* = 9). Significant differences (*p* < 0.05) are indicated with different letters (a–c), and were determined using one-way ANOVA followed by Tukey’s post hoc test.

HFD-induced obese mice exhibited notably elevated serum levels of ALT, AST, TG, total cholesterol, high-density lipoprotein (HDL) cholesterol, and low-density lipoprotein (LDL) cholesterol (Figure 2A–F). Following TLE treatment, ALT, AST, and TG levels remained within the normal range; however, total cholesterol, HDL cholesterol, and LDL cholesterol levels remained higher than those in the normal control group. Blood glucose concentrations increased significantly over time in the HFD group (Figure 2G,H). 

The increase in the activity of AST and ALT observed in the HFD group indicated liver damage. 

### 3.3. Effects of TLE on Gut Permeability

**Figure 3:**
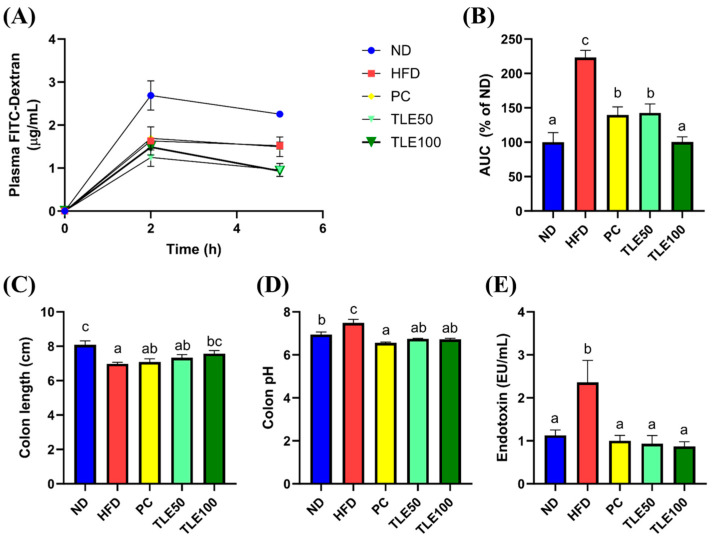
Effects of thyme leaf extract (TLE) on parameters reflecting the gut environment and on serum endotoxin levels in HFD-induced obese mice. (**A**) Levels of plasma fluorescein isothiocyanate (FITC)-dextran over 5 h; (**B**) area under the curve (AUC) for plasma FITC-dextran levels over time; (**C**) colon length (cm); (**D**) colon pH; (**E**) serum endotoxin levels. The data are expressed as mean ± standard error of the mean (*n* = 9). Significant differences (*p* < 0.05) are indicated with varying letters (a–c), and were determined using one-way ANOVA followed by Tukey’s post hoc test.

To assess the effect of TLE on intestinal permeability, we administered FITC-dextran and examined the area under the curve (AUC) for the plot of plasma FITC-dextran fluorescence over time. The blood FITC-dextran concentrations were significantly elevated in the HFD group, and were notably reduced upon TLE treatment (Figure 3A,B). The colon length was significantly shorter in the HFD group than in the ND group (Figure 3C). In mice treated with TLE, the colon length gradually increased, reaching levels similar to those in the normal control group. The colon pH was higher in the HFD group than in the normal group; however, after TLE treatment, the pH level resembled that of ND group (Figure 3D). Mice on a HFD demonstrated markedly elevated levels of serum endotoxin levels compared with mice fed a normal diet. Furthermore, TLE-treated mice showed reduced endotoxin levels comparable to those observed in the ND group (Figure 3E). 

### 3.4. Effect of TLE on the Expression of Tight Junction Proteins

**Figure 4:**
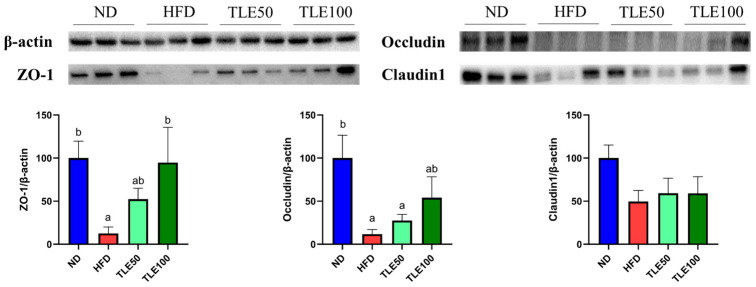
Effects of thyme leaf extract (TLE) on colon tight junction barrier function in high-fat diet (HFD)-induced obese mice. Expression levels of ZO-1, occludin, and claudin1 in the colon tissue of mice were analyzed. Band intensities are normalized against β-actin levels and data are expressed as mean ± SEM. Significant differences (*p* < 0.05) are indicated with different letters (a,b), and were determined using one-way ANOVA followed by Tukey’s post hoc test.

To assess the expression of tight junction proteins involved in the gut barrier function, we examined the expression of ZO-1, occludin, and claudin 1 (Figure 4). The expression of ZO-1 was significantly higher in the TLE 100 mg/kg group than in the HFD group. Although the level of occludin was increased compared with the HFD group, it exhibited a significant decrease compared with the ND group. For claudin 1, no significant differences were observed in any of the groups. These immunoblotting results show that TLE treatment has the potential to modulate the expression of ZO-1 (Figure 4).

### 3.5. Correlation between Metabolites and Serum Metabolic Disorder Parameters 

**Figure 5:**
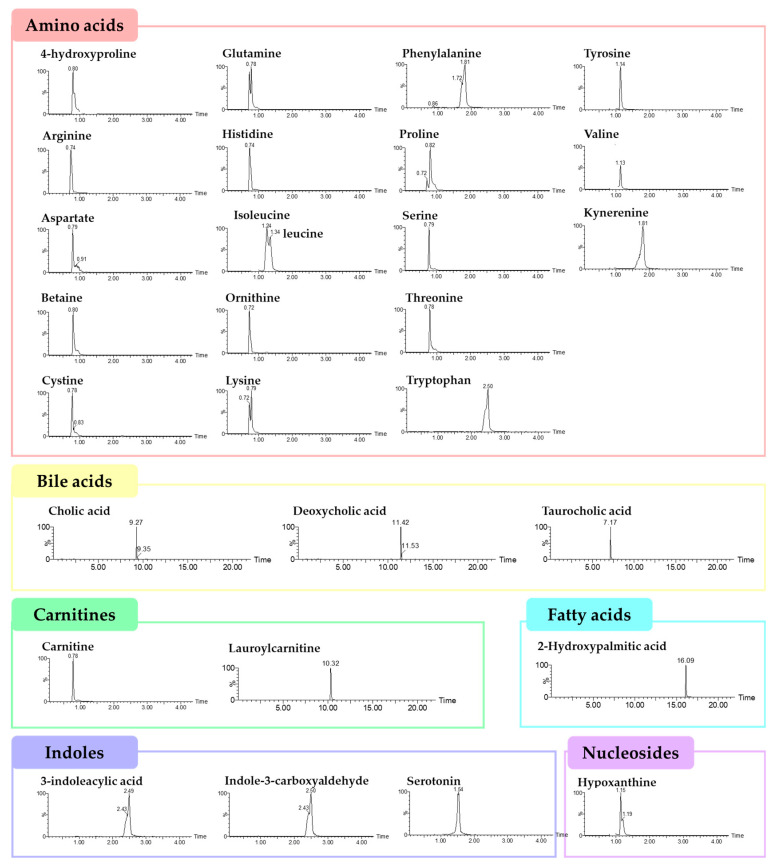
LC-MS/MS chromatograms of the thirty-one target analytes in the multiple reaction monitoring mode following pretreatment.

LC-MS/MS analysis was performed to assess the concentration of fecal metabolites of each group. A comprehensive quantitative analysis of 31 metabolites was conducted. Chromatograms of the analyte in the multiple reaction monitoring mode were obtained following pretreatment (Figure 5). 

**Figure 6:**
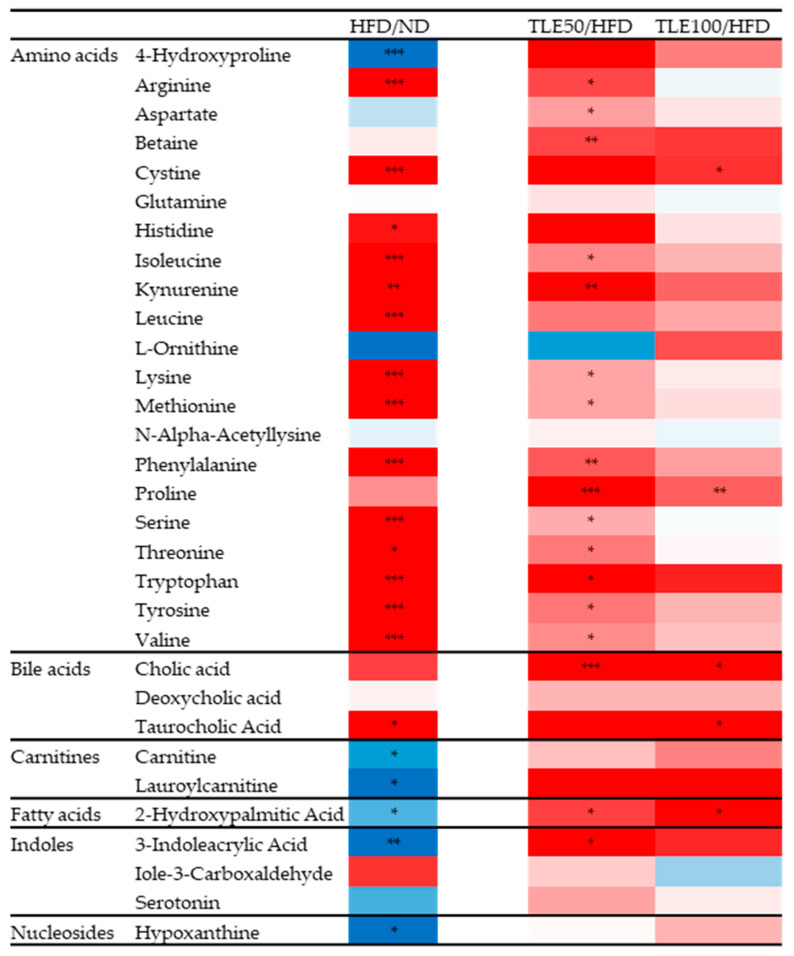
Heatmaps comparing HFD with the normal diet group, and HFD with the group administered TLE. The color ranges from deep red, indicating high abundance, to deep blue, indicating low abundance. * *p* < 0.05, ** *p* < 0.01, *** *p* < 0.001.

Differences in the 31 metabolites between the HFD, ND and TLE-treated groups were confirmed by comparing heatmaps (Figure 6). Based on the heatmap results, it was observed that in the HFD group, most amino acid metabolites were significantly increased, excluding 4-hydroxyproline. However, metabolites related to the carnitine pathway, hydroxypalmitic acid and 3-indoleacrylic acid, exhibited a significant decreasing trend in the HFD group. Particularly, 2-hydroxypalmitic acid and 3-indoleacrylic acid showed a significant increase in the TLE-treated group. 

**Figure 7:**
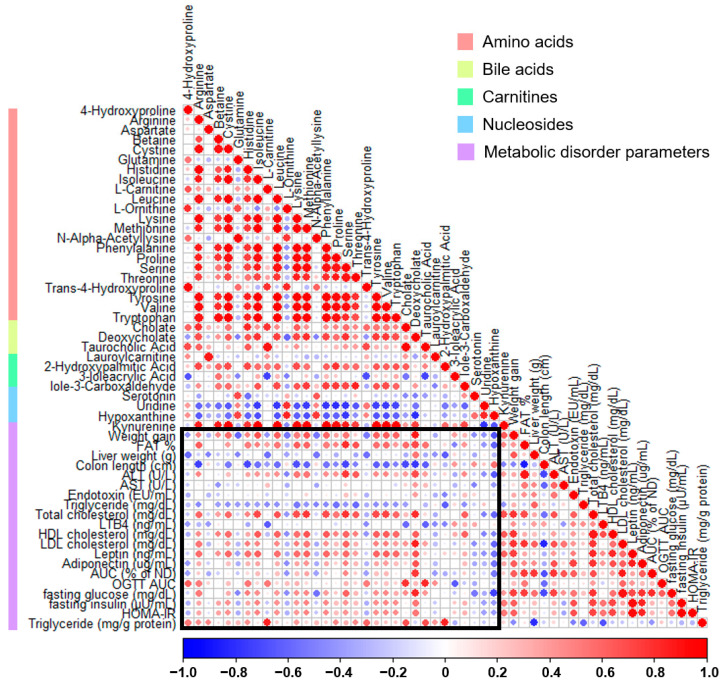
Correlation plot between fecal metabolites and metabolic disorder parameters in high-fat diet-induced obese mice treated with thyme leaf extract (TLE). The color scale indicates Pearson correlation coefficients, spanning from blue (indicating a negative correlation) to red (indicating a positive correlation). Metabolite types are indicated by the color bar on the left.

The association between fecal metabolites and parameters linked to metabolic disorders was assessed through Pearson’s correlation analysis (Figure 7). In the weight gain category, most metabolites related to amino acid pathways, except for glutamine and ornithine, were positively correlated. Conversely, the majority of amino acids and bile acid-related metabolites were negatively correlated with the colon length. In particular, a positive correlation was observed between various glucose-related parameters, HDL cholesterol, LDL cholesterol, and the gut permeability AUC associated with deoxycholic acid. In contrast, hypoxanthine was predominantly negatively correlated with various metabolic disorder parameters, and only the colon length showed a positive correlation.

## 4. Discussion

High-fat induced disorders of lipid metabolism accelerate metabolic syndromes, including obesity, hyperlipidemia, nonalcoholic fatty liver disease, and cardiovascular disorders [25], and are strongly linked to alterations in the gut microbiota [26]. Food intake has also been linked to protection against chronic conditions. Regular consumption of plant-based phytochemical-rich foods with health-promoting attributes may diminish the likelihood of chronic ailments in individuals [27]. Many studies have reported the effects of plant-derived extracts on human gut health, and their anti-obesity activities are well documented [28]. In this study, we investigated the anti-obesity and gut health-promoting properties of TLE in vivo.

Thyme is a significant botanical species with medicinal and culinary value. Researchers have shown considerable interest in the composition and biological effects of its polyphenolic extracts. Owing to the exceptional anti-inflammatory properties of thyme polyphenols and their remarkable probiotic activity, TLE contributes to the alleviation of ulcerative colitis [20]. Thymol, the primary component of TLE, exhibits anti-inflammatory properties [29]. Thyme extract also exhibits antibacterial [30], antiviral [31], antifungal [32], anticancer [33], antihypertensive [34], antioxidant [35], antiproliferative [36], and antinematode activities [37]. However, research on whether thyme extracts have anti-obesity and gut health-promoting effects has been scarce. We, therefore, investigated the potential benefits of thyme extract in addressing the problem of obesity and in promoting gut health.

We observed that administering TLE mitigated the weight gain caused by the HFD in mice without affecting their food consumption. Additionally, TLE treatment significantly decreased the levels of markers related to liver function, such as ALT, AST, and LDL cholesterol, while significantly increasing the HDL cholesterol levels. In particular, elevated levels of ALT and AST, linked to the heightened presence of alcohol dehydrogenase enzymes in the liver, are responsible for facilitating the conversion of alcohol into its respective aldehyde [38]. Examination of the liver tissue of mice with progressive hepatic injury after TLE treatment revealed their recovery to a normal state. This indicates that the TLE extract reduces hepatic damage caused by NaNO_2_ [39].

The gastrointestinal tract plays a critical role in nutrient absorption and immune protection, and the gut microbiota contributes to these functions through the fermentation of indigestible substances, vitamin synthesis, and involvement in the host’s immune responses [40]. Colon shortening is an indicator of colon impairment [41]. The gut microbiota modulates energy balance and fat accumulation. Tissue damage and mucosal inflammation can be assessed using markers such as gut permeability [42]. A HFD is known to change the gut microbiota composition and to increase intestinal permeability and inflammation [43]. Notably, mice raised in a germ-free environment demonstrated resistance to diet-induced obesity [44]. Key roles in preventing metabolic endotoxemia and preserving the integrity of the intestinal barrier are attributed to adherens junction proteins like ZO-1, occludin, and claudin [45]. Our results show that TLE supplementation improved the HFD-induced reduction in the expression of ZO-1 in the colon, and while the levels of FITC-dextran, a marker of intestinal permeability, significantly increased in the HFD group, treatment with TLE led to a significant reduction in gut permeability. This suggests that TLE administration enhances gut health by improving the expression of tight junction proteins, including ZO-1 [46].

The gut microbiota is closely associated with human health and diseases [47]. Functional assessment of the gut microbial community can be achieved through omics studies, including genomics, transcriptomics, proteomics, and metabolomics [48]. Analysis of fecal metabolites enables research on the host–microbiota interactions, providing insights into the dynamic interplay between the host and gut microbes [49]. 

The analysis of metabolites revealed contrasting trends in 2-hydroxypalmitic acid and 3-indoleacrylic acid between mice treated with TLE and those on a HFD. It was observed that these metabolites exhibited a pattern returning to normal levels. However, specific studies on the anti-obesity or gut health effects of these two metabolites could not be found. Yet, a comprehensive review indicated that overall, branched hydroxyl-fatty acids demonstrate anti-diabetic and anti-inflammatory effects [50]. After metabolism, the indole-3-propionic acid produced from 3-indoleacrylic acid improves blood glucose, suppresses inflammatory factors, corrects gut microbial imbalance, maintains intestinal barrier integrity, and inhibits intestinal immune responses [51]. Due to the lack of separate metabolite-specific studies, our findings suggest the need for further research on palmiticacid and indoleacrylic acid, metabolites identified in our study that may represent the effects of TLE.

Bile acids influence not only bile formation and fat absorption, but also processes such as inflammation, cell apoptosis, and hepatocyte necrosis [52]. A HFD elevates the concentration of deoxycholic acid in the gut while decreasing the secretion of interleukin-22 [53]. Bile acids play a significant role in the treatment of obesity, with studies confirming significantly elevated levels in obese individuals, suggesting their potential contribution to metabolic regulation following weight control [54]. This aligns with our findings that show a positive correlation between metabolic disorder parameters and deoxycholic acid. It underscores the importance of effective management of deoxycholic acid distribution, particularly in the context of a HFD and its mitigation. Moreover, studies have indicated a relationship among hypoxanthine concentration, NAFLD symptoms [55] and obesity [56], highlighting the significance of examining alterations in various metabolites. Most metabolites, with the exception of hypoxanthine, exhibited a negative correlation with colon length, which was linked to the regulation of the gut microbiota and restoration of the intestinal barrier function. Our study provides broad insights relevant for a comprehensive understanding of metabolic disorders for future research on the treatment and diagnosis of metabolic diseases by confirming the correlation between various metabolites and metabolic disorder parameters. However, owing to the current ambiguity in interpreting these relationships, further research is required to establish meaningful interconnections.

In this study, we investigated the anti-obesity and gut health-promoting effects of thyme extract. Treatment with 100 mg/kg TLE significantly reduced fat percentage. The significant decrease in the AUC for plasma FITC-dextran over time, used to assess gut permeability, in the TLE group suggests its potential as a beneficial substance for gut health.

## 5. Conclusions

Based on various findings regarding the anti-obesity and gut health-promoting properties of TLE elucidated in this study, it is evident that administering the extract to mice has beneficial effects on obesity and gut health. Clinical trials are required to assess how these effects translate in humans. Our study indicates that thyme exhibits promise as a natural therapeutic remedy for preventing and addressing obesity as well as other metabolic disorders.

## Figures and Tables

**Table 1:** Optimized mass spectrometer information for the simultaneous analysis.

Group	Compound	Precursor	Product	CE	RT (min)
Amino acids	4-Hydroxyproline	132.1	86.2	14	0.9
	Arginine	175.1	70.2	20	0.87
	Aspartate	134.1	74.1	14	0.93
	Betaine	118.1	59.1	16	0.95
	Cystine	241.1	152	12	0.88
	Glutamine	148.1	84.2	14	0.92
	Histidine	156.1	110.2	14	0.86
	Isoleucine	132.1	86.2	10	1.43
	Leucine	132.1	86.2	12	1.55
	L-Ornithine	133.1	70.2	14	0.87
	Lysine	147.1	84.2	14	0.84
	Methionine	150.1	104.1	12	1.23
	*N*-Alpha-Acetyllysine	189.3	84.1	20	0.84
	Phenylalanine	166.1	120.1	14	2.17
	Proline	117	71.1	14	0.94
	Serine	106	60.2	10	0.9
	Threonine	120	74.1	8	0.92
	Tryptophan	205.1	146.1	18	3.16
	Tyrosine	182.1	136.1	12	1.29
	Valine	118.1	72.2	10	1.25
	Kynurenine	209.1	146.1	18	2.23
Bile acids	Cholic acid	407.3	343.3	30	12.71
	Deoxycholic acid	391.3	345.2	28	14.52
	Taurocholic Acid	514.3	80.1	58	9.89
Carnitines	L-Carnitine	162.1	60.2	16	0.94
	Lauroylcarnitine	344.4	85	24	13.14
Fatty acids	2-Hydroxypalmitic Acid	271.3	225.2	20	17.83
Indoles	3-Indoleacrylic Acid	188.1	115.1	28	7.94
	Indole-3-Carboxaldehyde	146.1	118.1	14	6.33
	Serotonin	177.1	115.1	24	1.74
Purines	Hypoxanthine	137	110.1	20	1.29

## Data Availability

The data presented in this study are openly available. Data are available from a publicly accessible repository.
